# Trends in Patterns of Intermediate Uveitis in a Tertiary Institution in Singapore

**DOI:** 10.1371/journal.pone.0091533

**Published:** 2014-03-13

**Authors:** Helen Mi, Su L. Ho, Wee K. Lim, Elizabeth P. Y. Wong, Stephen C. Teoh

**Affiliations:** 1 Yong Loo Lin School of Medicine, National University of Singapore, Singapore, Singapore; 2 National Healthcare Group Eye Institute, Tan Tock Seng Hospital, Singapore, Singapore; 3 Eagle Eye Centre, Singapore, Singapore; Oregon Health & Science University, United States of America

## Abstract

**Purpose:**

The study aims to describe the characteristics and etiologic causes of intermediate uveitis (IU) patients seen by a tertiary eye center in Singapore over 8 years.

**Methods:**

This was a retrospective analysis of the clinical records of consecutive new cases of IU that presented to the uveitis subspecialty clinic from 2004–2011 at Tan Tock Seng Hospital. Data collected included demographics, clinical and laboratory findings. Diagnoses were based on standardized clinical history, ophthalmological examination and investigations.

**Results:**

There were 66 new cases of IU, comprising 5.7% of 1168 new uveitis patients. The median age of diagnosis was 40 years (mean 39.4±15.9), with largest subgroup of the patients in the age group of 41–60 years (36.4%). The majority was Chinese (57.6%), followed by Asian Indians (18.2%) and Malays (16.7%). The ethnicity distribution was dissimilar to our ethnic distribution in Singapore (p<0.001) with an increased incidence of IU in the Asian Indian population. Most were idiopathic (59.1%) in etiology, followed by tuberculosis (TB) (15.2%). Ocular complications developed in 21 patients (31.8%), with cystoid macular edema (CME) being the commonest (28.8%). Severe vitritis occurred in 9.1% of patients, and was significantly associated with TB-associated IU (p<0.001). There was a downward trend for the incidence of the proportion of IU patients over the total uveitis patients (p = 0.021), with Spearman’s rho of −0.786.

**Conclusions:**

Despite the downward trend, TB-associated IU was still of higher prevalence compared to less endemic areas, emphasizing the need for increased TB surveillance. A high index of suspicion for TB-associated IU is required in patients with severe vitritis. Comparisons with other countries revealed disparities in the IU etiologies, indicating possible geographical differences. Prevalence of known immune-mediated etiologies of IU is less compared to the western population. Our study also suggests a probable predisposition of the Singapore local Indian population for IU.

## Introduction

Intermediate uveitis (IU) is the form of uveitis least commonly associated with a systemic disorder [1]–[2], with its etiological patterns not well-described in the literature [2]–[5], especially in an Asian multi-racial population. The anatomical classification of IU was based on the criteria of the International Uveitis Study Group (IUSG) [6]. This classification was affirmed by the Standardization of Uveitis Nomenclature (SUN) Working Group, which defined IU as a subset of uveitis where vitreous is the major site of inflammation, with or without peripheral vascular sheathing and macular edema [7].

IU can be associated with systemic infectious diseases, or known immune-mediated illness such as sarcoidosis and multiple sclerosis (MS) [8]. Geographical, demographic and ethnic factors may influence its pattern [3]–[4], [9]. Improvement in diagnostic techniques may also change the apparent prevalence of etiological diagnoses [5]. Studies of the distribution of the various types and causes of uveitis are important in aiding the clinician in the appropriate and focused approach to investigation, diagnosis and management [10]. Comprehending the global variations in epidemiology of IU patterns is crucial for comparison of treatment practices, optimal assessment, management and complications of IU. By investigating trends of the etiologies of IU, patterns of various diseases in the population may also be identified and monitored.

We had previously described the clinical characteristics and changing trends in patterns of etiologies among anterior uveitis (AU) patients seen by the uveitis service of a tertiary eye care center in Singapore [11]. Currently, we aim to report the pattern of etiology trends and clinical characteristics in patients with IU seen similarly by the uveitis service at our center over an 8-year period.

## Materials and Methods

We retrospectively analyzed the case records of all consecutive new cases seen at the Uveitis and Ocular Inflammation service of Tan Tock Seng Hospital (TTSH), Singapore. Diagnosis of IU was made at presentation, and classification of IU was done according to the International Uveitis Study Group (IUSG) [6] recommendations and affirmed by SUN Working Group [7] criteria described earlier. To obtain diagnoses for specific ocular entities or systemic disease associations, all IU patients underwent a standardized clinical history, systemic review, complete ophthalmological examination and standardized laboratory investigations. Laboratory tests, included a complete blood count (CBC), erythrocyte sedimentation rate (ESR), C-reactive protein (CRP), syphilis screen consisting of Venereal Disease Research Laboratory (VDRL), and treponemal serology (syphilis IgG) tests, immune markers consisting of rheumatoid factor (RF), anti-double-stranded deoxyribonucleic acid (DNA) (anti-dsDNA), anti-neutrophil cytoplasmic antibodies (ANCA), chest radiograph, *Mantoux* skin test and/or tuberculosis (TB) interferon gamma release assay (IGRA) (T-SPOT.*TB*, Oxford Immunotec Ltd, U.K.). Further ancillary investigations were performed based on clinical presentation and signs where necessary, including polymerase chain reaction (PCR) DNA analysis, serologic IgG analysis, and human leukocyte antigen (HLA) typing and vitreous biopsy. Magnetic resonance imaging was considered and performed if patients exhibited neurological symptoms or signs suggestive of MS.

The criteria for the most commonly diagnosed diseases in this cohort were as follows. Presumed TB-associated uveitis was diagnosed when there was exclusion of other known etiologies of uveitis, suggestive clinical history and signs, supportive investigations such as positive *Mantoux* reaction, TB IGRA and chest radiograph findings, and response to empirical anti-tuberculosis treatment, and in some, evidence of *Mycobacterium tuberculosis* or its DNA in ocular fluid/tissues [12]–[13]. Fuchs heterochromic iridocyclitis (FHI) was diagnosed when there are uveitis with characteristic white stellate keratic precipitates, diffuse iris stromal atrophy with variable iris pigment epithelial atrophy and iris color changes [14]. All other systemic diseases were diagnosed according to current diagnostic criteria, with referral to the respective specialists. The term idiopathic was used for cases in which the intraocular inflammation was not characteristic of a recognized uveitic entity or could not be attributed to a specific underlying systemic disease. FHI was considered to be idiopathic. Severe vitritis was defined as IU with grade >2+ haze [5], [15]. Masquerade syndrome was excluded if patients responded to standard immunosuppressive therapy.

Data collected included the demographics of the patients, including gender, race, age at presentation, year at presentation and eye(s) affected, as well as clinical and laboratory results. Other systemic co-morbidities and medical history were also noted. All descriptive data were entered into a computerized database system for descriptive analysis, and analyzed with IBM SPSS Statistics (version 19, IBM Corp, New York, USA) and R version 2.14.1. Fisher’s Exact test was used to determine any association between etiologies with ethnicity, as well as etiologies with complications. Bonferroni correction was done for the comparison between the local ethnic distribution in Singapore and the ethnic distribution in our study, with statistical significance defined to be p<0.0167. Categorical-type data were analyzed with Pearson Chi-Square goodness-of-fit test, specifically the comparison of ethnic distribution between the data collected and the Singapore population. Spearman’s rho was used to check for linear tendencies of etiologies and IU patients from 2004–2011. A p-value less than 0.05 was considered to indicate statistical significance.

The investigation adhered to the tenets of the Declaration of Helsinki. The study protocol obtained Institutional Review Board (IRB) approval from the National Healthcare Group Domain Specific Review Board (NHG-DSRB), with waiver of informed consent.

## Results

### Demographics

Our study included 66 patients with newly diagnosed IU, comprising 5.7% of 1168 new patients diagnosed with uveitis between years of 2004 and 2011. In comparison with the other classifications of uveitis from 2004–2011, there were 795 AU patients (68.1%), 177 posterior uveitis patients (15.2%) and 130 panuveitis patients (11.1%). With the exception of IU, majority of all the other uveitis patients were males, and had unilateral disease. The profile of the Singapore population and detailed demographic profile of patients and laterality, stratified based on the various classification of uveitis, is listed in Table 1.

**Table 1:** Comparison of demographic details of uveitis patients and the Singapore population.

Etiology	IU		AU		Posterior uveitis		Panuveitis		Singapore (Thousands)	
**Number of patients/people (n, %)**	66	(5.7)	795	(68.1)	177	(15.2)	130	(11.1)	3789.3	
**Gender**										
Male	30	(45.5)	484	(60.9)	97	(54.8)	71	(54.6)	1868.2	(49.3)
Female	36	(54.5)	311	(39.1)	80	(45.2)	59	(45.4)	1921.1	(50.7)
**Laterality**										
Bilateral	37	(56.1)	157	(19.7)	67	(37.9)	36	(27.7)	–	–
**Race**										
Chinese	38	(57.6)	564	(70.9)	98	(55.4)	90	(69.2)	2808.3	(74.1)
Malay	11	(16.7)	91	(11.4)	29	(16.4)	18	(13.8)	506.6	(13.4)
Asian Indians	12	(18.2)	73	(9.2)	21	(11.9)	8	(6.2)	349.0	(9.2)
Others	5	(7.6)	67	(8.4)	29	(16.4)	14	(10.8)	125.3	(3.3)
**Age Groups**										
0–20	11	(16.7)	24	(3.0)	11	(6.2)	8	(6.2)	897.5	(23.7)
21–40	23	(34.8)	225	(28.3)	76	(42.9)	41	(31.5)	1131.5	(29.9)
41–60	24	(36.4)	329	(41.4)	70	(39.5)	43	(33.1)	1199.4	(31.7)
>60	8	(12.1)	217	(27.3)	20	(11.3)	37	(28.5)	560.8	(14.8)

**Figure 1:**
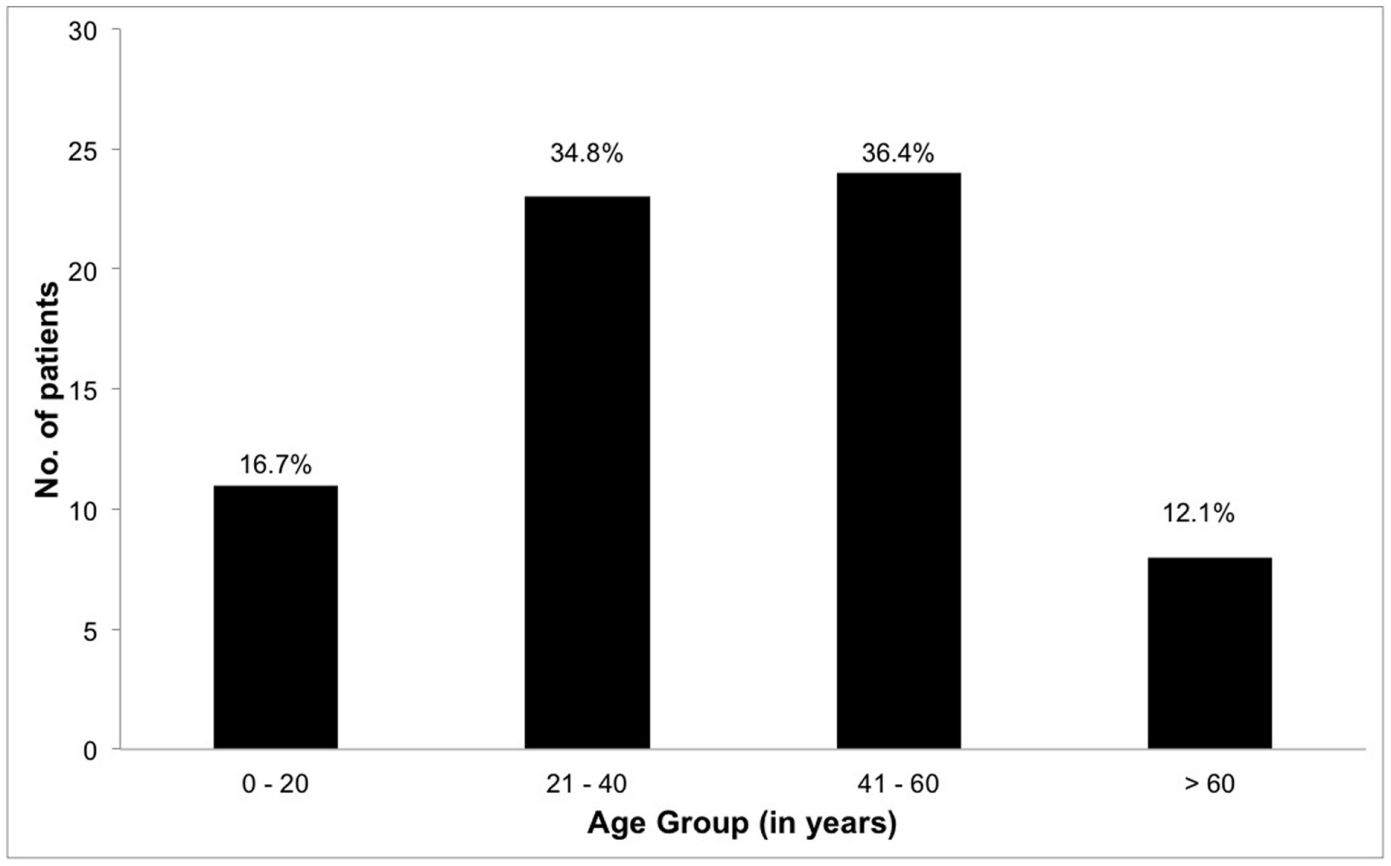
Age distribution of IU patients, showing that largest subgroup of the patients was in the age group of 41–60 years.

There were 30 males (45.5%) and 36 females (54.5%). The disease was bilateral in 37 patients (56.1%). The majority of patients were of Chinese ethnicity (n = 38, 57.6%), followed by Asian Indians (n = 12, 18.2%), and Malays (n = 11, 16.7%). The median age at diagnosis was 40.0 years (mean 39.4±15.9 years, range 6–71 years). The largest subgroup of the patients were in the age group of 41–60 years (36.4%), followed by 21–40 years (34.8%), then 0–20 years (16.7%), and finally >60 years (12.1%) (Figure 1). Three patients (4.5%) were in the pediatric age group, which is defined as younger than 16. Age distribution and demographic profile of the IU patients are shown in Figure 1 and Table 2. In total, there were 37 patients (56.1%) who had bilateral IU. The proportion of bilateral IU was 21 (58.3%), 6 (42.9%) and 10 (71.4%) for the idiopathic IU, infective IU and known immune-mediated IU patients respectively. The detailed demographic profile of patients and laterality, stratified based on etiology, is listed in Table 2.

**Table 2:** Demographic profile of patients.

Etiology	Idiopathic		Infective		Immune-mediated		Masquerade	
**Number of patients (n, %)**	36		14		14		2	
**Gender**								
Male	15	(41.7)	6	(42.9)	9	(64.3)	0	(0.0)
Female	21	(58.3)	8	(57.1)	5	(35.7)	2	(100.0)
**Race**								
Chinese	23	(63.9)	6	(42.9)	8	(57.1)	1	(50.0)
Malay	3	(8.3)	4	(28.6)	3	(21.4)	1	(50.0)
Asian Indians	7	(19.4)	2	(14.3)	3	(21.4)	0	(0.0)
Others	3	(8.3)	2	(14.3)	0	(0.0)	0	(0.0)
**Laterality**								
Bilateral	21	(58.3)	6	(42.9)	10	(71.4)	0	(0.0)

**Figure 2:**
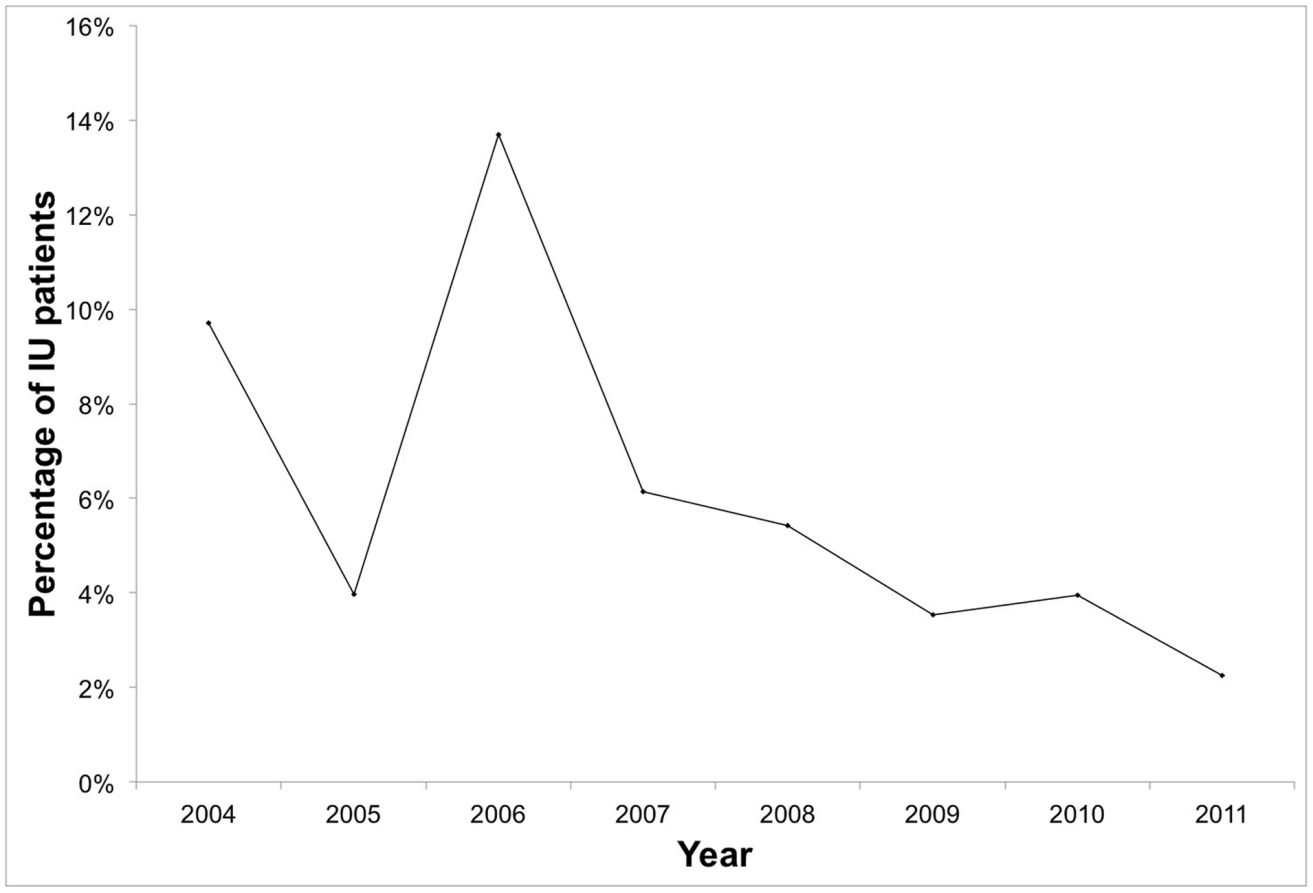
Proportion of IU patients over the total number of uveitis patients. This showed a statistically significant (p = 0.021) downward trend, with Spearman’s rho of −0.786. This is possibly due to an increasing trend of the total number of uveitis patients, while the incidence of IU patients had been generally stable, in comparison.

There was a general statistically significant (p = 0.021) downward trend for the proportion of IU patients over the total uveitis patients (except 2006) in our tertiary referral eye center, with Spearman’s rho (ρ) of −0.786 (Table 3 and Figure 2).

**Table 3:** Incidence and proportion of IU and total uveitis patients.

Year	No. of IU patients	No. of uveitis patients	Percentage of IU/uveitis patients (%)
2004	10	103	9.7
2005	4	101	4.0
2006	10	73	13.7
2007	10	163	6.1
2008	11	203	5.4
2009	9	255	3.5
2010	7	177	4.0
2011	5	222	2.3

### Etiology of Intermediate Uveitis

**Figure 3:**
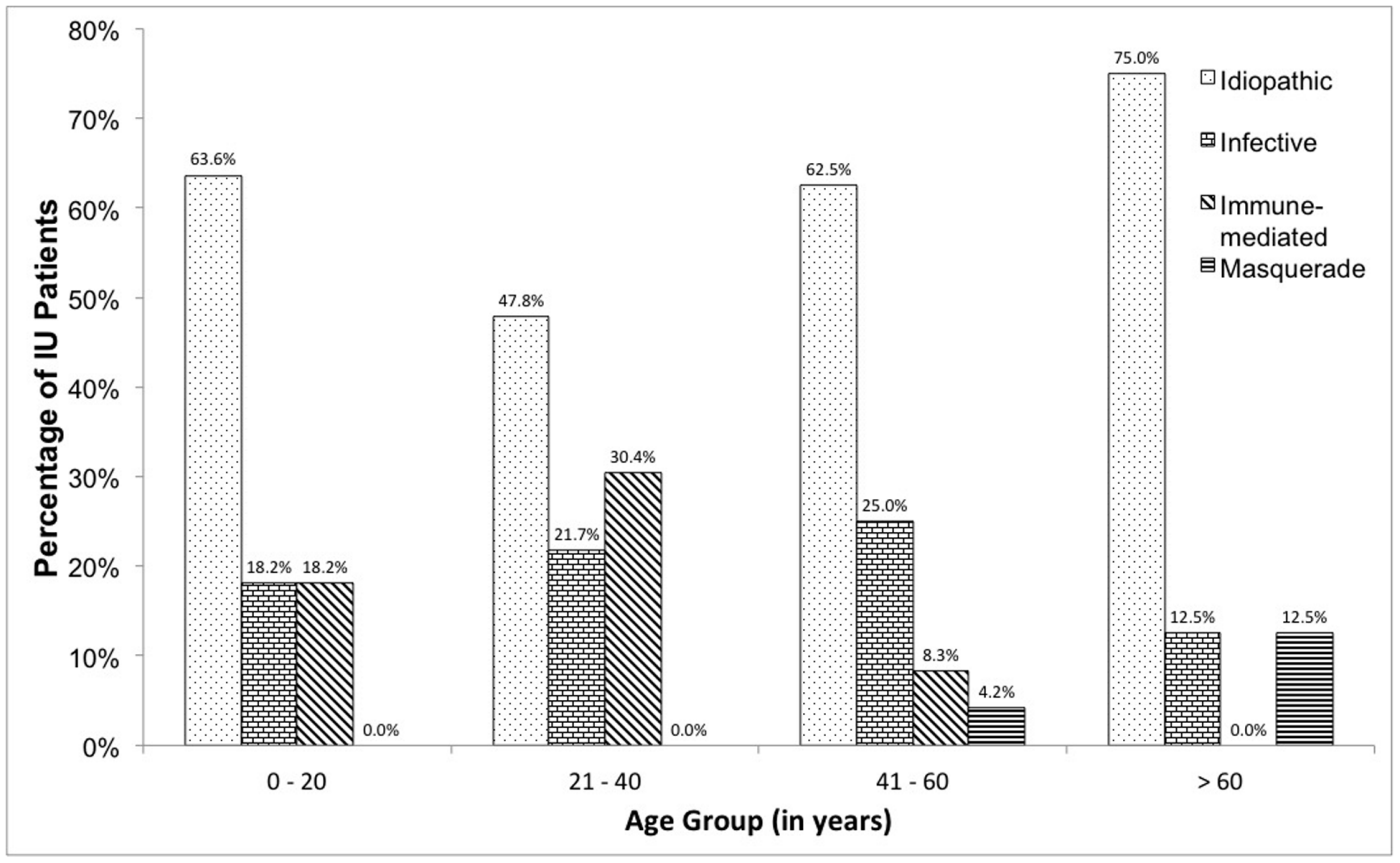
Etiology distribution of IU patients stratified by age groups. Idiopathic IU was the commonest diagnosis across all age groups, but there were a high proportion of IU patients with known immune-mediated etiology in the 21–40 age group (30.4%).

Majority of the cases were non-infectious (n = 52, 78.8%). Of the 66 patients over 8 years, 39 (59.1%) were idiopathic, 14 (21.2%) had an underlying infective cause, and another 11 (16.7%) had a known immune-mediated etiology. Masquerade syndromes (ocular lymphoma) were diagnosed in 2 patients (3.0%). Idiopathic IU was the commonest diagnosis across all age groups, but there were a high proportion of IU patients with known immune-mediated etiologies in the 21–40 age group (n = 7, 30.4%). Detailed trending of age groups by etiology is shown in Table 4 and Figure 3. Using the Fisher’s Exact test, there was no statistically association between age groups and the etiology categories (p = 0.410).

**Table 4:** Classification of etiology by age groups.

	Age Groups (years)								Total	
	0–20		21–40		41–60		>60			
**No. of patients (n)**	**11**		**23**		**24**		**8**		**66**	
**Idiopathic (n, %)** *	**7**	**(63.6)**	**11**	**(47.8)**	**15**	**(62.5)**	**6**	**(75.0)**	**39**	**(59.1)**
**Infective (n, %)** *	**2**	**(18.2)**	**5**	**(21.7)**	**6**	**(25.0)**	**1**	**(12.5)**	**14**	**(21.2)**
TB	2	(18.2)	4	(17.4)	4	(16.7)	0	(0.0)	10	(15.2)
Syphilis	0	(0.0)	1	(4.3)	0	(0.0)	1	(12.5)	2	(3.0)
HSV†	0	(0.0)	0	(0.0)	1	(4.2)	0	(0.0)	1	(1.5)
HIV	0	(0.0)	0	(0.0)	1	(4.2)	0	(0.0)	1	(1.5)
**Immune-mediated (n, %)** *	**2**	**(18.2)**	**7**	**(30.4)**	**2**	**(8.3)**	**0**	**(0.0)**	**11**	**(16.7)**
Sarcoidosis	0	(0.0)	2	(8.7)	2	(8.3)	0	(0.0)	4	(6.1)
MS	0	(0.0)	2	(8.7)	0	(0.0)	0	(0.0)	2	(3.0)
Behcet	0	(0.0)	2	(8.7)	0	(0.0)	0	(0.0)	2	(3.0)
AS	1	(9.1)	0	(0.0)	0	(0.0)	0	(0.0)	1	(1.5)
Psoriasis‡	0	(0.0)	1	(4.3)	0	(0.0)	0	(0.0)	1	(1.5)
JIA	1	(9.1)	0	(0.0)	0	(0.0)	0	(0.0)	1	(1.5)
**Masquerade (n, %)** *	**0**	**(0.0)**	**0**	**(0.0)**	**1**	**(4.2)**	**1**	**(12.5)**	**2**	**(3.0)**

*expressed as percentage of patients within age group.

† HSV was diagnosed with a positive polymerase chain reaction test.

‡ Psoriasis was diagnosed based on a clinical diagnosis of IU and dermatologic manifestations diagnosed as psoriasis by a dermatologist.

Abbreviations: TB, tuberculosis; HSV, Herpes simplex virus; HIV, human immunodeficiency virus; MS, multiple sclerosis; AS, ankylosing spondylitis; JIA, juvenile idiopathic arthritis.

**Figure 4:**
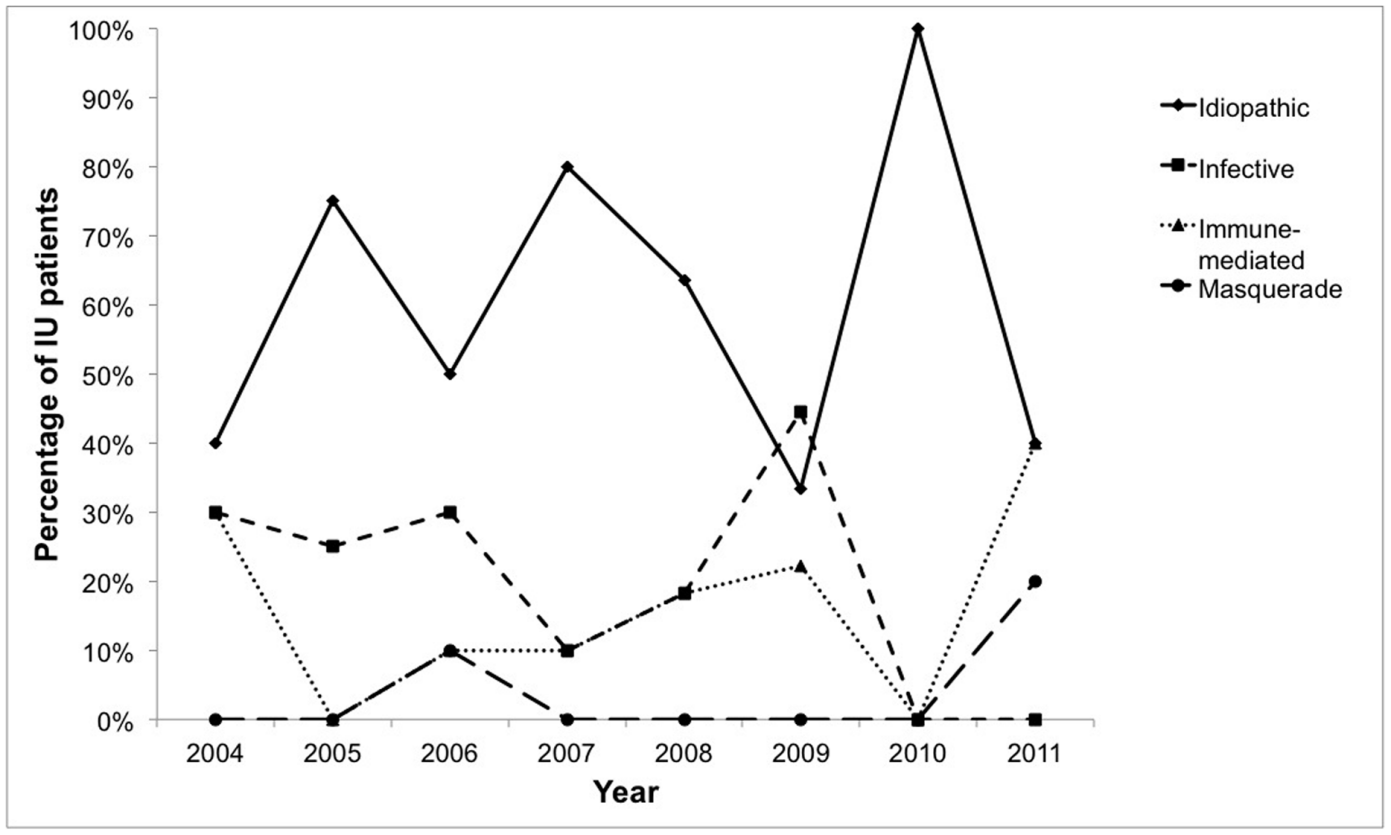
Stratification of etiologies of IU from 2004 to 2011. There was a general downward tendency for the infective etiologies, with Spearman’s rho of −0.566.

There was an even spread of newly diagnosed IU patients over 2004–2007, with a subsequent general downward tendency noted from 2008–2011. Looking at the patterns of the various etiologies over 2004–2011, we noticed a general downward tendency for the infective etiology category (Spearman’s rho (ρ) = −0.566, p = 0.143). The detailed pattern of etiologies over 2004–2011 is shown in Figure 4.

Of the 14 patients with infective etiology, the most common in our population was presumed TB (n = 10, 15.2%), followed by syphilis (n = 2, 3.0%). Eight of the 10 patients (80.0%) diagnosed with presumed TB were tested positive for TB IGRA, and 5 of these patients (50.0%) had positive *Mantoux* skin reactions of ≥8 mm. Three of these patients (30.0%) had both positive IGRA and *Mantoux* skin reaction. All of the patients diagnosed with presumed TB had suggestive clinical history and signs, while none of the 10 patients had positive chest radiograph findings or presence of TB DNA in ocular fluid/tissues. Among the patients with infective etiology, majority had unilateral IU (n = 8, 57.1%). Of the 11 patients with known immune-mediated etiologies, the most common etiology was sarcoidosis (n = 4, 6.1%), followed by Behcet’s disease (n = 2, 3.0%) and MS (n = 2, 3.0%). Among the patients with known immune-mediated etiologies, majority had bilateral IU (n = 10, 71.4%).

The most common form of IU among Chinese (n = 25, 65.8%) and Asian Indians (n = 8, 66.7%) was idiopathic, while that in Malays (n = 4, 36.4%) was infective (Table 2). Using the Fisher’s Exact test, there was no statistically significant association between ethnicity and etiology categories (p = 0.468). The most common form of IU among males (n = 15, 50.0%) and females (n = 21, 58.3%) was idiopathic. Males had a higher incidence of known immune-mediated IU (n = 9, 30.0%) compared to females (n = 5, 13.9%). Using the Pearson Chi-Square test, there was no statistically significant association between gender and etiology categories (p = 0.271).

In total, 21 IU patients (31.8%) developed complications, with cystoid macular edema (CME) being most common (n = 19, 28.8%). This was followed by chronic glaucoma (n = 4, 6.1%). Two (3.0%) patients with associated ocular hypertension were eventually diagnosed with masquerade syndrome secondary to intraocular lymphoma. There were 6 patients (9.1%) with severe vitritis associated with decreased visual acuity, of which 83.3% were diagnosed with TB uveitis. Using the Fisher’s exact test, there is a statistically significant (p<0.001) association between severe vitritis and TB-associated IU.

### Treatment

Fifty (75.8%) patients with IU required treatment. Corticosteroids were used in all treatments. Fifteen (22.7%) received topical therapy alone, 4 (6.1%) received systemic therapy alone, 11 (15.2%) received topical and systemic therapy, and 10 (15.2%) required adjuvant periocular steroids. Immunosuppressive treatment was used only in non-infective etiologies of IU, and 10 patients (15.2%) received concurrent immunosuppressive therapy. Seven patients (10.6%) received only 1 type of concurrent immunosuppressive therapy and 3 (4.5%) received 2 types of concurrent immunosuppressive therapy, on top of corticosteroid therapy. Immunosuppressive therapy used include that of methotrexate (n = 3, 4.5%), azathioprine (n = 6, 9.1%), cyclophosphamide (n = 1, 1.5%), tacrolimus (n = 2, 3.0%), and mycophenolate mofetil (n = 4, 6.1%). Presumed TB patients received full antibiotic treatment as per the WHO guidelines.

IU patients with CME, chronic glaucoma and severe vitritis were treated with systemic and/or periocular steroids. These patients made up 38.0%, 12.0% and 8.0% of treated patients respectively.

## Discussion

The overall incidence of IU is the lowest anatomic type among uveitis occurrences in published Asian, European and Northern American studies, with incidence ranging from 5–20% [2], [5], [16]–[20]. We observed this similar trend in our center comprising only 5.7% of uveitis patients seen at our tertiary service. In our population, IU was most common in the Chinese population (57.6%), followed by Asian Indians (18.2%), and Malays (16.7%), which was statistically dissimilar (p<0.001) to our ethnic distribution in Singapore (Chinese 74.1%, Malays 13.4%, Asian Indians 9.2%) [21], suggesting a probable predisposition of the Singapore local Indian population for IU. This is contrasted with our previous findings in AU, which was commonest in the Chinese population (69.8%), followed by Malays (13.2%), and Asian Indians (11.4%) [11]. Our center had a slightly higher prevalence amongst females (54.5%), similar to other published studies (USA 64.0%, Japan 55.0%, India 54.9%) [6]–[7], [22]. The age of presentation amongst our IU patients were in the fourth decade, which was similar to other Asian studies [5], [15], [23]. They were also older compared to patients with uveitis in general, which was reportedly common in the second-third decade [3], [20], [22], [24]–[25]. We also had a low incidence of uveitis among our pediatric age group (4.5%), which is similar to other published studies (5–10%) [26]. We noticed a statistically significant (p = 0.021) downward trend for the proportion of IU patients over the total uveitis patients, with Spearman’s rho (ρ) of −0.786. This is likely due to a decreasing trend of the IU patients while the incidence of the total number of uveitis patients had been generally stable in comparison in our tertiary eye center, especially between 2008 and 2011 (Table 3). The decreasing trend in our center could also be attributed to possible changes in referral patterns due to better management of the etiologies of IU in the primary care setting.

From our study, patients with IU had a relatively high incidence of an infective etiology, particularly TB (15.2%), especially in our age group of 30–40 years. This was consistent with our tuberculosis incidence rates among our population, with the highest incidence between the 4^th^ to 7^th^ decade [27]. This could be also due to a demographic difference or a later presentation of symptoms due to decreased awareness. Countries endemic with infective diseases such as TB have a much higher rate of presumed TB etiologies compared to our cohort, with our incidence of 15.2% compared to 46.7% (India) [4]. Of note, there was no significant (p>0.999) association between the Asian Indian IU patients in our study and TB, using the Fisher’s Exact test. The likely cause could be that the Asian Indian patients in our study are mostly local patients who went through the legally mandatory Bacillus Calmette-Guérin (BCG) vaccination at birth. However, our incidence (15.2%) was higher than other less endemic areas, such as Japan and USA, with an incidence of 6.9% [5] and 7.0% [20] respectively. This correlates with World Health Organization (WHO) estimates of TB prevalence in Singapore to be of 35 per 100,000, compared to 185 per 100,000 in India, and only 4.1 per 100,000 in the USA. TB-associated uveitis is more commonly associated with posterior uveitis (choroiditis) or panuveitis than IU [10], [13], [28]–[30]. Severe vitritis is also reported to be significantly associated with latent TB uveitis in predominantly posterior segment inflammation [10]. Our study showed statistically significant association between severe vitritis and TB-associated IU (p<0.001). In our previously reported AU cases, there was a relatively low incidence of TB [11]. This may further indicate that TB uveitis possibly presents more commonly as IU or posterior uveitis than AU. This highlights the need for an increased index of suspicion of TB uveitis in IU patients with severe vitritis in TB endemic countries.

Most cases of IU were idiopathic. This was a similar finding in many other published studies in Asian and Caucasian nations, ranging from 70–90% in Africa, Europe and USA [2], [9], [16]–[17], [20], [24], [31] and 42.3% in Japan [5]. Despite the increased use of investigational tools in determining the etiology of IU, this worldwide consistency suggests that typically, specific systemic etiologies of IU could not be determined. However, it could be possible that IU is commonly due to a local pathological process, rather than a systemic one. It is expected that with the advances in knowledge and diagnostic techniques, the proportion of idiopathic uveitis cases would decline over time [32]. Our incidence of 54.5% is more similar to that of Japan, compared to Africa, Europe and USA, consistent with genetic and geographical differences between Asians and Caucasians. These differences were also evidenced in other etiologies [5], [9]. MS was strongly associated with IU in western population, ranging from 14.8%–16.2% [33]–[34], compared to 3.0% in our study. The prevalence of MS in South East Asia is low, with an incidence of only 2–3 per 100,000 [35], compared to 196 per 100,000 [36] amongst European and American Caucasians. MRI was thus not routinely performed as part of our workup for patients with intraocular inflammation in view of the low prevalence of MS in South East Asia. In Japan, there was a much higher prevalence of immune-mediated etiologies of IU, seen in Vogt-Koyanagi-Harada (VKH) disease (10.1% versus 0%), sarcoidosis (9.5% versus 6.1%), and Behcet’s disease (5.8% versus 3.0%) [5].

The limitations of this study are the retrospective nature, and the relatively smaller number of patients. There is the problem of referral or selection bias, as our tertiary referral institution sees the more severe cases, resulting in the data reflecting a select subset of our population and not representative of the general population of Singapore. Nevertheless, IU is a relatively uncommon disease, and this data was obtained over a period of 8 years. Also, our tertiary institution has no bias in terms of referral patterns towards any particular age group or ethnicity. Moreover, the results of our study are consistent with other Asian studies and as such, this suggests that these results are representative.

In summary, our results suggest a statistically significant downward trend for the proportion of IU patients over the total uveitis patients in our tertiary referral eye center. In TB endemic countries, a high index of suspicion for TB-associated uveitis is required in patients with associated severe vitritis. Ocular complications developed in about one-third of cases with CME being the most common. Comparisons with other countries showed disparities indicating possible genetic and geographical differences in the etiology of IU.
